# Supplementary material for: Modeling enamel matrix secretion in mammalian teeth
Source: PLoS Comput Biol. 2019 May 29;15(5):e1007058. doi: 10.1371/journal.pcbi.1007058 (PMC6541238; doi:10.1371/journal.pcbi.1007058)
Supplement: S1 Fig — Whereas both the diffusion-limited simulation and geometric extrapolation of Fig 4 approximate the amount of real enamel, only the diffusion-limited simulation reproduces the length of the perimeter of the real surface. The drop in enamel perimeter in the diffusion-limited simulation towards the cusp tip relative to empirical data is due to the horizontal simulations not capturing the relatively round EDJ tip. (PDF) [file pcbi.1007058.s001.pdf]

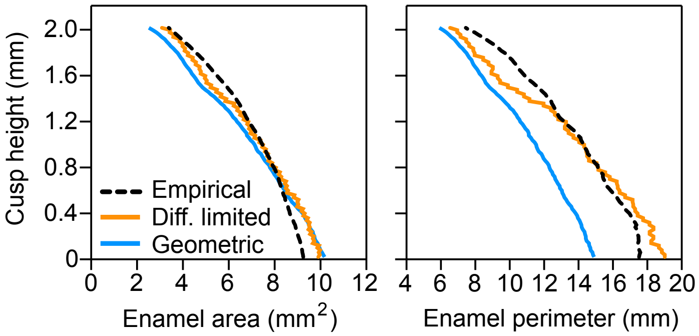

S1 Fig. Enamel area and surface perimeter in horizontal slices of a pig cusp. Whereas both the diffusion-limited simulation and geometric extrapolation of Fig. 4 approximate the amount of real enamel, only the diffusion-limited simulation reproduces the length of the perimeter of the real surface. The drop in enamel perimeter in the diffusion-limited simulation towards the cusp tip relative to empirical data is due to the horizontal simulations not capturing the relatively round EDJ tip.
