# Supplementary material for: Modeling enamel matrix secretion in mammalian teeth
Source: PLoS Comput Biol. 2019 May 29;15(5):e1007058. doi: 10.1371/journal.pcbi.1007058 (PMC6541238; doi:10.1371/journal.pcbi.1007058)
Supplement: S1 Table — Node density denotes the number of nodes along the longest axes in the regular triangular mesh. ‘Border’ is the proportional size of the margins surrounding the EDJ shape. 'Base’ sets the y-axis position of the base in simulations using a sink. All EDJ simulations are run with Neumann domain boundaries, except for the simulations using basal sink for which we use mixed boundaries (Dirichlet/Neumann), with a fixed time step 0.001. All parameters are dimensionless and their absolute values have no meaning. (PDF) [file pcbi.1007058.s002.pdf]

S1 Table. EDJ conversion and simulation parameters. Node density denotes the number of nodes along the longest axes in the regular triangular mesh. ‘Border’ is the proportional size of the margins surrounding the EDJ shape. 'Base’ sets the y-axis position of the base in simulations using a sink. All EDJ simulations are run with Neumann domain boundaries, except for the simulations using basal sink for which we use mixed boundaries (Dirichlet/Neumann), with a fixed time step 0.001. All parameters are dimensionless and their absolute values have no meaning.

	Level set conversion			Simulation				
Simulation	Node density	Border	Base	Nutrient diffusion rate	Nutrient require-ment	Inter-facial tension	Back-ground nutrient	Itera-tions (N)
**Synthetic shape**								
Diff. limit.	320	0.65	-	1.0	1.5	0.0025	25	16
Excess nutr.	320	0.65	-	1.0	1.5	0.0025	300	6
No tension	320	0.65	-	1.0	1.5	0.0	25	16
**Pig molar**								
2D molar	200	0.01	-	1.0	1.5	0.002	40	9
3D cusp	200	0.1	-	1.0	1.5	0.005	40	16
**Human molar**								
Diff. limit.	400	0.1	0.165	1.0	2.2	0.00001	75	4
Excess nutr.	400	0.1	0.165	1.0	2.2	0.005	160	2
**Orangutan molar**								
Diff. limit.	400	0.1	0.165	1.0	2.2	0.00001	30	7
