# Supplementary material for: Modeling enamel matrix secretion in mammalian teeth
Source: PLoS Comput Biol. 2019 May 29;15(5):e1007058. doi: 10.1371/journal.pcbi.1007058 (PMC6541238; doi:10.1371/journal.pcbi.1007058)
Supplement: S2 Table — The DB values are calculated for the binary outlines of the EDJs and enamel surfaces. The background nutrient production values for the excess background nutrient, weak and strong diffusion-limited effect simulations are 160, 75, and 30, respectively. (PDF) [file pcbi.1007058.s003.pdf]

S2 Table. Box-counting dimensions calculated for human and orangutan molar teeth. The D_B values are calculated for the binary outlines of the EDJs and enamel surfaces. The background nutrient production values for the excess background nutrient, weak and strong diffusion-limited effect simulations are 160, 75, and 30, respectively.

Sample	Mean	Min	Max	s.d.
**EDJ**				
Human	1.001	0.951	1.093	0.0356
Orangutan	1.007	0.959	1.090	0.0304
**Real enamel surface**				
Human	1.010	0.966	1.102	0.0323
Orangutan	1.077	1.033	1.155	0.0297
**Excess background nutrient**				
Human	1.000	0.956	1.083	0.0305
Orangutan	1.008	0.971	1.095	0.0268
**Weak diffusion-limited effect**				
Human	1.010	0.965	1.091	0.0287
Orangutan	1.022	0.988	1.114	0.0274
**Strong diffusion-limited effect**				
Human	1.045	0.993	1.123	0.0281
Orangutan	1.085	1.046	1.172	0.0290
