# Supplementary material for: Modeling enamel matrix secretion in mammalian teeth
Source: PLoS Comput Biol. 2019 May 29;15(5):e1007058. doi: 10.1371/journal.pcbi.1007058 (PMC6541238; doi:10.1371/journal.pcbi.1007058)
Supplement: S1 Appendix — (PDF) [file pcbi.1007058.s004.pdf]

S1 Appendix

Model background. The classical Stefan problem considers phase transition of undercooled liquid to solid [1, 2]. We adopt the same terminology for our purposes: We refer to both the dentin and the enamel matrix secreted by ameloblasts as "solid", and the space into which the ameloblast front expands as "liquid". Note that these terms are purely for convenience: We do not make any assumptions about the mechanical properties of the matter and consequently, the terms solid and liquid are essentially arbitrary. Finally, we refer to the diffusing substance required for the growth as "nutrient".

Model principles. Consider a system depicted in Fig. 1, where Ω_1 and Ω_2 are spaces occupied by liquid and solid phases of a substance, respectively. An influx of nutrient from the liquid phase is required for the solid to grow, and the growth takes place at the interface $\Gamma(t)$ separating Ω_1 and Ω_2. Denote the nutrient concentration in $\Omega = \Omega_1 \cup \Omega_2 \cup \Gamma$ by $u(x,t)$, and suppose the nutrient diffuses according to Fick's law of diffusion, that is, the flux is $\mathbf{q} = -\alpha \nabla u$, where α is the diffusion coefficient. Coupled with the conservation of mass and a background source term $f(x,t)$, we have

$$\frac{\partial u}{\partial t} = \alpha \nabla^2 u + f \qquad \text{in } \Omega. \tag{1}$$

In our simulations we only consider a source term f that is a constant step function such that

$$f(x,t) = \begin{cases} \beta, & x \in \Omega_1, \\ 0, & x \in \Omega_2 \cup \Gamma, \end{cases} \tag{2}$$

with $\beta \in \mathbb{R}^+$. In other words, there is a background nutrient source in the liquid domain and none in the solid domain or the interface.

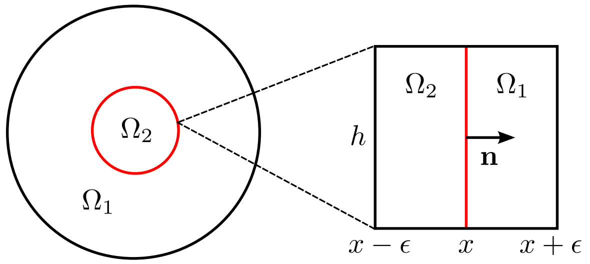

Figure 1. Schematic of the interface growth domain. Ω_1 denotes the liquid phase, Ω_2 the solid phase.

Depending on the situation, we use either pure Neumann (horizontal simulations) or mixed Dirichlet/Neumann (vertical simulations with a sink, see tooth specific methods) boundary conditions on the outer domain boundary $\partial\Omega = \partial\Omega^1 \cup \partial\Omega^2$, that is,

$$\frac{\partial u}{\partial \mathbf{n}} = 0 \text{ on } \partial\Omega^1 \quad \text{and} \quad u = B \text{ on } \partial\Omega^2,$$

where in the case of non-empty $\partial\Omega^2$ we may for example have $B = 0$ at one side of Ω (sink) and $B = 1$ at the opposite side (source). Pure Neumann simulates a system where the growth takes place in a closed chamber with the domain boundaries acting only as barriers.

We initialize the concentration $u(x,t)$ as

$$u(x,0) = \begin{cases} C_0, & x \in \Omega_1, \\ 0, & x \in \Omega_2 \cup \Gamma, \end{cases}$$

where $C_0 \in \mathbb{R}^+$ in the case of pure Neumann boundaries, and a steady-state solution of (1) in the case of mixed boundaries.

Next, we need a condition to describe the growth of the interface Γ. Without loss of generality, consider a small patch of width h along the interface, as shown in Fig. 1, sufficiently small so that the curvature of the interface can be ignored. Assuming the growth of the interface is irreversible we write

$$x - \epsilon = \Gamma(t) < \Gamma(t + \Delta t) = x + \epsilon.$$

Let L be the amount of nutrient required for growth in a unit of space, then

$$\begin{aligned}[\text{Total nutrient consumed}] &= [\text{Area}] \times L \\ &= \big[\Gamma(t+\Delta t) - \Gamma(t)\big] h \times L.\end{aligned}$$

Total nutrient consumed is equal to the amount of nutrient diffusing into the interface minus the nutrient diffusing out, thus

$$\begin{aligned}&\big[\Gamma(t+\Delta t) - \Gamma(t)\big] h \times L \\ &= h \int_t^{t+\Delta t} -\alpha \big[\mathbf{q}_{in} - \mathbf{q}_{out}\big] \cdot \mathbf{n}\, dt \\ &= h \int_t^{t+\Delta t} -\alpha \big[\nabla u(x+\epsilon, t) - \nabla u(x - \epsilon, t)\big] \cdot \mathbf{n}\, dt,\end{aligned}$$

where note that $\mathbf{q}_{in}$ is interpreted as the flux from Ω_1 to the interface, and similarly $\mathbf{q}_{out}$ as the flux from the interface to Ω_2, thus the signs are reversed. Dividing both sides by Δt and h, and taking the limit with respect to time gives

$$\begin{aligned}\frac{d\Gamma}{dt} L &= \lim_{\Delta t \to 0} \frac{\Gamma(t+\Delta t) - \Gamma(t)}{\Delta t} L \\ &= \lim_{\Delta t \to 0} \frac{1}{\Delta t} \int_t^{t+\Delta t} \alpha \big[\nabla u(x+\epsilon, t) - \nabla u(x-\epsilon, t)\big] \cdot \mathbf{n}\, dt \\ &= \alpha \big[\nabla u(x+\epsilon, t) - \nabla u(x-\epsilon, t)\big] \cdot \mathbf{n},\end{aligned}$$

where we interpret the gradients as one-sided differentials near Γ. Thus we have a jump condition on the interface

$$\frac{d\Gamma}{dt} L = VL = \alpha \left[\frac{\partial u_1}{\partial \mathbf{n}} - \frac{\partial u_2}{\partial \mathbf{n}}\right], \tag{3}$$

where u_1, u_2 denote that the derivates on Γ should be taken within Ω_1 and Ω_2, respectively. In the context of the classical Stefan problem, condition (3) with a sign reverse and separate diffusion coefficients for two phases is commonly known as the Stefan condition. The main difference here with respect to the Stefan problem is that whereas in the Stefan problem it is assumed that latent

heat must flow out of the interface in order for the growth to take place, we assume that there is a positive net flow of nutrients into the interface.

Finally, we need an interface condition for $u(x,t)$ at Γ. Without interfacial tension, we could simply set $u(x,t) = 0$, but since we assume the interface resists curvature similar to the surface tension, we set

$$u(x,t) = \epsilon_c \kappa, \qquad\qquad x \in \Gamma,$$

where ϵ_c is the tension coefficient and κ the local interface curvature. In the classical Stefan problem the sign of κ would be negative.

To summarize, the full system we solve is: Find u and Γ such that

$$\begin{cases} \dfrac{\partial u}{\partial t} = \alpha \nabla^2 u + f & \text{in } \Omega, \\ VL = \alpha \left[\dfrac{\partial u_1}{\partial \mathbf{n}} - \dfrac{\partial u_2}{\partial \mathbf{n}} \right] & \text{on } \Gamma, \\ u(x,0) = C_0 & \text{in } \Omega_1, \\ u(x,0) = 0 & \text{in } \Omega_2, \\ u(x,t) = \epsilon_c \kappa & \text{on } \Gamma, \\ \dfrac{\partial u}{\partial \mathbf{n}}(x,t) = 0 \text{ on } \partial\Omega^1 \quad \text{and} \quad u(x,t) = B \text{ on } \partial\Omega^2, \end{cases} \tag{4}$$

where $f := f(x,t)$ is the source function (2) and $\partial\Omega^1, \partial\Omega^2$ denote the subsets of boundary $\partial\Omega$.

Numerical implementation. Our numerical solver for system (4) follows the finite element method (FEM) implementation in [3], with minor modifications for the present problem type. For completeness, we describe the main aspects of the numerical solver in the following sections. We do not derive the weak forms of the equations here, for details on obtaining those see [3, 6]. Because our focus is on the empirical applications, we do not discuss the numerical properties of implementation in length, aside from basic validation to demonstrate the correctness of the implementation. While the FEM implementation implies the possibility of arbitrary element geometries, we have chosen here to discretize the domain Ω as a regular triangular mesh in order to minimize grid effects on the simulation behavior. For alternative, finite difference -based solvers of diffusion-limited growth, see [1, 2].

We use the level set method [5] for tracking the moving boundary in system (4). Briefly, the interface Γ is given implicitly as the zero of a signed distance function

$$\phi(x,t) = \begin{cases} +\mathrm{dist}(x, \Gamma(t)), & x \in \Omega_1, \\ 0, & x \in \Gamma, \\ -\mathrm{dist}(x, \Gamma(t)), & x \in \Omega_2. \end{cases} \tag{5}$$

After obtaining the interface velocity V from (3), the velocity is extended to whole domain, after which the interface position can be updated by solving an advection equation on ϕ. We define ϕ at the mesh nodes, from which we reconstruct the interface position for both computation and visualization purposes by remeshing the mesh with temporary nodes and edges denoting the zero of ϕ, as shown in Fig. 2.

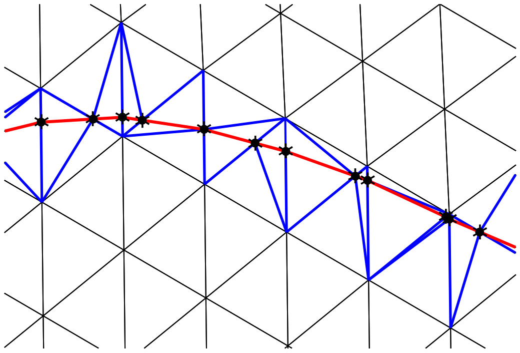

Figure 2. Interface reconstruction by temporary remeshing near the interface. Black asteriks denote the interface nodes interpolated from the values of $\phi(x, t)$ at the neighboring mesh nodes, red edges the discretized interface, and blue edges the supporting edges to maintain the triangular mesh.

An outline of the algorithm for solving system (4) is presented in Algorithm 1. In the following each step of the algorithm is overviewed, and references to the corresponding code implementations are given.

Algorithm 1 Matrix secretion

1: Initialize the computational domain.
2: Compute interface velocity at the interface Γ.
3: Extend the interface velocity field V to the whole domain.
4: Update the interface position by advecting ϕ.
5: Construct temporary interface-conforming mesh.
6: Reinitialize ϕ to a signed distance function.
7: Compute curvature κ at Γ, set interfacial tension.
8: Update the nutrient distribution.
9: While $t < T$, go to 2.

Initialization. To initialize the level set function (and thus the interface), we use digitized real EDJ shapes: The landmarks of each cusp are connected to form a closed loop of edges. This set of edges is then superimposed onto the computational mesh, after which the level set function is initialized based on the edge-edge crossings between the landmark edges and the computational mesh edges. Unlike the rest of the implementation, this step is implemented in Python (see *coordinates_to_levelset.py*). The rest of the mesh initialization on Matlab's end is implemented in *Init_mesh.m*.

The EDJ interior, denoted by Ω_2, is initialized as having zero nutrient concentration. The exterior Ω_1 is either initialized with a constant positive concentration with Neumann boundaries, or with a ramp concentration between mixed Dirichlet/Neumann boundaries. The ramp is obtained by finding the steady-state solution of (1) with the given Dirichlet boundaries. Finally, to avoid steep gradients at the interface, the initial concentrations are smoothed slightly with the presence of the interface by solving (1). For implementation details, see *Init_distribution.m*.

Computing interface velocity. We compute the interface velocity V in (3) for given growth param-

eter L over the interface edges, thus we can utilize element gradients for the nutrient field u, which are readily available from the FEM framework through the gradients of the global basis functions. For implementation details, see *Stefan_condition.m.*

Velocity extension. Given edge velocities V at the interface, we extend the velocity field to the full domain Ω by solving the Helmholtz equation separately in Ω_1 and Ω_2 with

$$\begin{aligned} \nabla^2 u - ku &= 0 && \text{in } \Omega_1, \\ \frac{\partial u}{\partial \mathbf{n}} &= V_n && \text{on } \Gamma, \end{aligned}$$

and

$$\begin{aligned} \nabla^2 u - ku &= 0 && \text{in } \Omega_2 \\ \frac{\partial u}{\partial \mathbf{n}} &= -V_n && \text{on } \Gamma, \end{aligned}$$

where V_n is the interface velocity in the direction of the normal to the interface. The Neumann boundary gives the correct velocity at the interface and almost correct velocity in a neighborhood of the interface. Changing signs of the Neumann condition between Ω_1 and Ω_2 makes the extended velocity continuous over the interface. The extension is not sensitive to the choice of k; in our implementation we have set $k = 1$.

The rationale for the velocity extension technique is that when we set the Neumann condition on the interface equal to the interface velocity, then

$$V_n = \frac{\partial u}{\partial \mathbf{n}} = \nabla u \cdot \mathbf{n} = \nabla u \cdot \frac{\nabla \phi}{|\nabla \phi|}$$

on the interface, and more generally for the extended (normal) velocity F holds

$$F = \nabla u \cdot \frac{\nabla \phi}{|\nabla \phi|} \tag{6}$$

away from the interface, as the gradient of u changes only gradually when moving away from the interface, and so the velocity can be assumed to be approximately correct in a neighborhood of the interface.

Fig. 3*B* shows an example of the velocity extension on a disk in symmetric surroundings. For implementation details, see *Velocity_extension_Helmholtz.m.*

Updating interface position. Following the standard level set method, given the extended velocity (6), we update the interface position indirectly by solving the advection equation

$$\frac{\partial \phi}{\partial t} + F|\nabla \phi| = 0 \qquad \text{in } \Omega, \tag{7}$$

with the natural boundary conditions (Neumann) on the domain boundary. We solve (7) with a variant of the implicit Euler method: Let M denote the mass matrix and K^n the advection matrix with velocity at step n on the static mesh nodes, then we solve with time step δ for the discretized level set coefficients ϕ_h^{n+1}

$$M\left(\frac{\phi_h^{n+1} - \phi_h^n}{\delta}\right) + K^n \phi_h^{n+1} = 0,$$

or after rearranging

$$\phi_h^{n+1} = (M + \delta K^n)^{-1} M \phi_h^n, \tag{8}$$

which we solve iteratively using the Matlab's *mldivide* method, as computing the inverse on the right-hand side directly would be excessively expensive. Note that in the standard implicit Euler method the matrix K is assembled using the velocity at step $n + 1$, thus requiring extrapolation. However, since in general the interface position evolves only gradually and so $K^n \approx K^{n+1}$, method (8) can be said to approximate the standard implicit Euler.

For implementation details, see *Interface_growth.m.*

Constructing interface-conforming mesh. A temporary interface-conforming mesh including the exact interface position is constructed by first creating new nodes along the interface-crossing edges where $\phi(x) = 0$, followed by retriangulation of the interface-crossing elements with the new nodes to maintain a correct triangular mesh (Fig. 2). For implementation details, see *Level_set.m.*

Reinitialization. To maintain the level set function as a distance function, which is needed for correct interface evolution, we reinitialize ϕ at every iteration by explicitly computing the distances from mesh nodes to the interface Γ. Away from the interface, we compute

$$\phi(x) = \min_p |p - x|, \qquad\qquad p \in \Gamma. \tag{9}$$

To improve accuracy within a narrow band around the interface, we compute orthogonal projection distances to the interface edges: For each node position x in a neighborhood of Γ, find

$$\phi(x) = \min_{(a,b)\in\Gamma} |x - [k(a - b) + b]|, \tag{10}$$

for those edges (a, b) for which

$$k = \frac{(x - b) \cdot (a - b)}{|a - b|^2} \in [0, 1].$$

For implementation details, see *Level_set.m.*

Computing curvature. Curvature κ in (4) for interfacial tension is computed as

$$\kappa = \nabla \cdot \mathbf{n} = \nabla \cdot \left(\frac{\nabla \phi}{|\nabla \phi|} \right),$$

which is readily computed in our FEM framework using the numerical gradient operator (see the source code). For better numerical stability, we compute the curvature only at the static mesh nodes, then linearly interpolate the curvature values at the interface nodes: Let n_1 and n_2 be the nodes of an edge crossed by the interface, then we set the curvature at the interface node n_i as

$$\kappa(n_i) = (1 - t)\kappa(n_1) + t\kappa(n_2),$$

where

$$t = \frac{\phi(n_1)}{\phi(n_1) - \phi(n_2)}.$$

For implementation details, see *Curvature_interpolate.m*.

Updating nutrient distribution. To update the nutrient distribution u in (4) we need to include all mesh nodes in the computation, including the interface, in order to accurately capture the process.

Denote the set of interface nodes and boundary nodes with Dirichlet condition by $\mathcal{B}$, and the set all nodes in which to solve the system by $\mathcal{I}$. Note that the sets are disjoint and that $\mathcal{B} \cup \mathcal{I}$ is the set of all computational nodes. We denote the submatrix of K containing rows R and columns C by $K(R, C)$.

Let M be the mass matrix and K the stiffness matrix. The basic linear system to solve for Eq. (1) is

$$M \frac{du_h}{dt} = -\alpha K u_h + M f.$$

where u_h is the vector of discrete solution coefficients.

Considering the values of u on the outer boundary and the interface nodes, we have

$$M(\mathcal{I}, \mathcal{I}) \frac{du_h}{dt}(\mathcal{I}) = -\lambda(K(\mathcal{I}, \mathcal{I}) u_h(\mathcal{I}) + K(\mathcal{I}, \mathcal{B}) u_h(\mathcal{B})) + M(\mathcal{I}, \mathcal{I}) f(\mathcal{I}).$$

To avoid repetition, we drop explicit mention of indices $\mathcal{I}$ and only write the indices if they are something other than $\mathcal{I}$. Rearranging and applying a first-order implicit method with time step δ gives

$$u_h^{n+1} = u_h^n - \delta M^{-1} \big[\alpha (K u_h^{n+1} + K(\mathcal{I}, \mathcal{B}) u_h^n(\mathcal{B})) - M f \big]$$

or after rearranging

$$u_h^{n+1} = \big[M + \delta \alpha K \big]^{-1} \big[M u_h^n + \delta (M f - \alpha K(\mathcal{I}, \mathcal{B}) u_h^n(\mathcal{B})) \big]. \tag{11}$$

Note that in (11) the boundary/interface contributions are computed using u_h^n; this makes our scheme essentially a first-order IMEX scheme [4]. As in the case of advection, we solve (11) using the Matlab's *mldivide*. For implementation details, see *Solve_heat.m*.

Validation. We investigated the validity of our numerical implementation by simulating a setting for which the classical Stefan problem has a known exact solution, the Frank-sphere exact test [2]. In short, the idea is to show that the radius of a disk at melting temperature surrounded by undercooled liquid converges towards the known value as the numerical resolution of the simulation is increased, and the disk should maintain its shape without surface tension.

We set the computational domain as a $\Omega = [-2, 2] \times [-2, 2]$ regular triangular mesh. The level set initialized so that there is initially (at $t = 1$) a disk of radius $s_0 = 0.25$ centered at the origin. The temperature is then initialized (at $t = 1$) with

$$T(s) = \begin{cases} 0, & s \leq s_0, \\ T_\infty \Big(1 - \frac{F(s)}{F(s_0)}\Big), & s > s_0, \end{cases}$$

where $T_\infty = -0.05709187113307$ is the temperature at the spatial infinity and

$$F(s) = \int_{s^2/4}^{\infty} \frac{e^{-t}}{t} \, dt.$$

Fig. 3*A* shows the initial disk disk shape at $t = 1$ and the final disk shape at time $t = 3$, demonstrating the maintenance of the circular shape. Fig. 3*B* shows the velocity field in the neighborhood of the disk after performing the velocity extension. Fig. 3*C* demonstrates the convergence of the disk radius towards the exact solution $r_{exact} = s_0\sqrt{t}$ as a function of the spatial resolution. Time step for all simulations is set as $\delta = 2 \times \Delta x$, where Δx is the spatial step size in the regular grid. Surface tension ϵ_c is kept at zero for all simulations.

The test is initialized in *Init_circle.m* and *Frank_init.m* for domain and temperature, respectively.

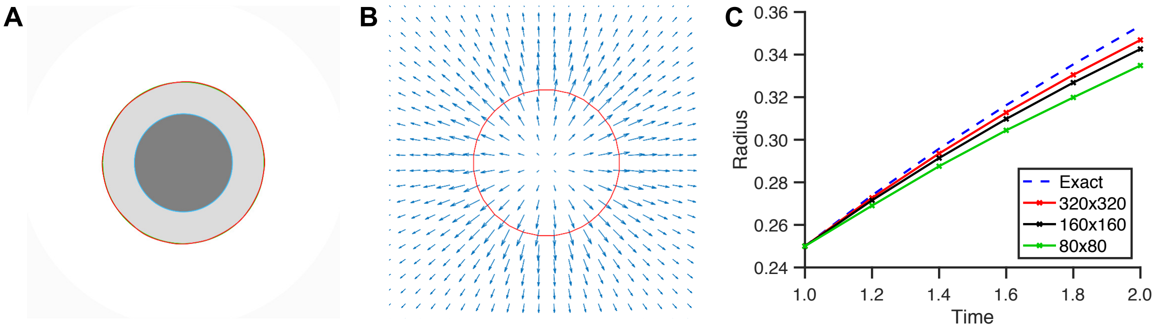

Figure 3. Validation of the numerical implementation. (***A***) Computed Frank-sphere solution (red) at time $t = 3$, resolution 320×320, with blue indicating the initial interface and green the perfect circle for reference (mostly covered by the red). (***B***) Extended velocity field. (***C***) Convergence of the disk radius to the exact solution.

References

1. Chen, S., Merriman, B., Osher, S. and Smereka, P., A simple level set method for solving Stefan problems. *J. Comp. Physics* **135**, 8–29 (1997).

2. Chen, H., Min, C. and Gibou, F., A numerical scheme for the Stefan problem on adaptive Cartesian grids with supralinear convergence rate. *J. Comp. Physics* **228**, 5803–5818 (2009).

3. Häkkinen, T. On Free Boundary Problems. MSc thesis, Aalto University (2016).

4. Ruuth, S.J., Implicit-explicit methods for reaction-diffusion problems in pattern formation. *J. Math. Biol.* **34**, 148–176 (1995).

5. Osher, S. and Sethian, J.A., Fronts propagating with curvature-dependent speed: algorithms based on Hamilton-Jacobi formulations. *J. Comp. Physics* **79**, 12–49 (1988).

6. Braess, D., Finite elements: Theory, fast solvers, and applications in solid mechanics. Cambridge University Press (2007).
